# Cutaneous Metastases of Solid Tumors: Demographic, Clinical, and Survival Characteristics

**DOI:** 10.7759/cureus.19970

**Published:** 2021-11-28

**Authors:** Isabel Betlloch-Mas, Tamara Soriano-García, Ignacio Boira, Juan Carlos Palazón, Gloria Juan-Carpena, Jose N Sancho-Chust, Eusebi Chiner

**Affiliations:** 1 Dermatology, Hospital General Universitario de Alicante, Alicante, ESP; 2 Pulmonology, Hospital Universitario San Juan Alicante, Alicante, ESP

**Keywords:** internal tumors, metastatic carcinoma, lung carcinoma, breast carcinoma, cutaneous metastasis

## Abstract

Background: Cutaneous metastasis (CM), while uncommon, is usually an indicator of poor prognosis. With cancer patients living longer, the incidence of CM has increased, which justifies its analysis.

Objectives: The objective of this study was to carry out a descriptive study of CM diagnosed for 18 years in a dermatology department of a tertiary care hospital and to assess the epidemiological, clinical, and histological variables that condition them, as well as data on their survival and prognosis.

Methods: We performed a descriptive study of cases of CM diagnosed over 18 years in the dermatology department of a tertiary referral hospital analyzing the following variables: patient age and sex, site of primary neoplasm, pathochronology, survival time, histological findings, immunohistochemical markers, the anatomical area affected, the clinical appearance of the metastasis, therapeutic plan, and existence of metastases in other regions. We checked normal distribution using the Kolmogorov-Smirnov test and then compared the quantitative variables using the Student's t-test (unpaired samples), Mann-Whitney test (non-normal distribution), analysis of variance (ANOVA; for more than two groups), and categorical variables using the chi-square or Fisher’s exact test.

Results: We included 37 cases (20 men and 17 women), of whom 32 had died. The mean age was 62 ± 15 years. CM detection was defined early in 8% of cases, synchronous in 32%, and metachronous in 60%. The most frequent primary tumor sites were lungs (24%), breasts (21%), and bladders (11%). Most metastases were solitary. The most frequent locations for CM were the scalp, trunk, armpits, and groin. Most lesions had a nodular presentation (81%). Squamous cell carcinoma and adenocarcinoma showed the same frequency in lung cancer CM. Breast cancer leading to CM was the most common invasive ductal carcinoma. The most aggressive cases, with the worst survival, originated in lung neoplasms. Therapeutic management for most patients involved surgery in combination with other procedures. The only difference detected between the lung and breast cancer CM was the predominance of lung tumors in men (89%) and breast tumors in women compared with metastases from other sites; breast cancer CM manifested more frequently as plaques and less frequently as nodules (p < 0.05) and was less frequently associated with multisystemic metastasis. In lung cancer CM, time from tumor diagnosis to CM occurrence was shorter (p < 0.01) and multisystemic metastasis was more frequent than in CM of other tumors.

Conclusions: CM tends to affect patients aged above 60 years and arises predominantly from lung cancer in men and breast cancer in women. The most typical locations are the chest and scalp, and the appearance is usually nodular. Survival after CM detection is low, particularly in lung cancer CM.

## Introduction

Cutaneous metastasis (CM) refers to the spread of malignant solid tumors to the skin [1]. Recognizing this presentation can be crucial for the diagnosis of unknown neoplasms [2]. CM normally results from the lymphatic and hematogenous spread of cancer cells, though direct invasion and iatrogenesis are also possible causes [3].

CM accounts for only 2% of all skin neoplasms, with an incidence of 0.7% to 9% [1], though this rate is on the rise as a result of cancer patients living longer [2]. Men are more commonly affected than women (37% vs 6%) [3,4], and most patients are aged between 50 and 70 years [5]. The rare cases in children are usually associated with neuroblastoma or leukemia, the most frequent tumors in this population [5]. The time-lapse between tumor and CM detection is estimated at 33 months, though CM is sometimes the first manifestation of a tumor [6]. Metastasis of a tumor to the skin usually indicates a poor prognosis, in some cases confirming an advanced stage of cancer [4]. Survival after detection is low, with half of the patients dying within six months. Consequently, therapy tends to be palliative. There is hope, however, in the form of novel treatments such as immunotherapy [2].

Examining a CM can help to diagnose the primary tumor as the two have similar histological patterns [1,7]. Up to six morphological patterns of CM have been described: nodular, infiltrative, diffuse, intravascular, top-heavy, and bottom-heavy [5]. Specific immunohistochemical markers can be used to define the primary tumor [4,7,8].

Malignant melanoma is the neoplasm that most frequently metastasizes to the skin, but it is excluded from most studies as only lesions found more than 2 cm from the primary tumor and beyond the first lymph node station are considered distant metastases. Lung cancer is the second most common source of metastasis to the skin and the leading source of CM in men. In women, breast cancer shows the highest frequency of CM [2]. Other tumors with a predilection for the skin include carcinoma of the renal cells, genitourinary apparatus, and colon/rectum; head and neck cancer; and some hematologic malignancies (primarily acute myeloid leukemia) [4].

CM location depends on the primary tumor [6]. Metastatic lesions tend to manifest close to the primary site, predominantly on the trunk or abdomen [2,4,9]. Some primary tumors have more specific metastatic locations; one example is renal cell carcinoma, which normally metastasizes to the scalp, although this location has also been reported in breast and gastrointestinal tumors, among others [8,9]. Colorectal carcinoma CM can appear close to the site of tumor removal or colostomy, or sometimes on the perineum. CM on surgical scars has also been reported in breast cancer patients. Lung cancer usually spreads to areas of skin above the diaphragm (7,8). Head and neck carcinoma tends to spread by direct extension, meaning CM lesions are usually close to this anatomical area [2]. Immune status may also affect the location of CM; the most susceptible sites are those with a higher density of regulatory T cells and few CD8+ T cells [10].

The most typical clinical presentation of CM is solitary or multiple nodules [5]. Morbidity is considerable as the painful, bleeding, and deforming lesions directly affect the patients’ quality of life. There are peculiar but not pathognomonic forms of CM that should provoke a high level of suspicion. Telangiectatic carcinoma, carcinoma erysipeloides, carcinoma en cuirasse, and alopecia neoplastica [11,12] are forms that are mainly associated with breast cancer [2,6]. Breast adenocarcinoma can also manifest as a characteristic form of CM affecting the nipple and areola, known as Paget’s disease [12]. Lung cancer has been associated with a phenomenon known as “clown nose”: a reddish or purplish and sometimes painful metastatic nodule located on the tip of the nose [13]. CMs in colorectal carcinoma and some gastrointestinal, hepatocellular, and pancreatic carcinomas have a characteristic umbilical form known as the Sister Mary Joseph nodule [2,4].

The differential diagnosis of skin lesions suggestive of CM should include other pathologies with benign or malignant dermatological involvement and systemic diseases such as vasculitis. The prognostic implications of an early diagnosis are especially important in breast cancer and melanoma, but clinicians should also suspect CM resulting from an underlying visceral neoplasm.

In our study, we aimed to review the demographic, clinical, and histological characteristics of CM cases treated in our hospital as well as the associated survival rate and prognosis. This article was previously presented as a meeting abstract at the 2021 Annual European Academy of Dermatology and Venereology (EADV) Scientific Meeting in September 2021.

## Materials and methods

We conducted a retrospective study of patients treated in the Dermatology Department of Alicante University General Hospital, Alicante, Spain, from July 2002 to June 2020. This hospital serves an estimated population of 262,962 inhabitants. We included all patients diagnosed with CM and excluded patients with melanoma, non-melanoma skin cancer, or hematological malignancies (leukemia) as well as those with incomplete data in their medical records.

We analyzed the demographic variables (sex and age) and clinical variables (location of primary neoplasm, affected system, time from diagnosis of neoplasm to detection of metastasis, time from diagnosis of CM to death of patient, findings of histological and immunohistochemical analysis of the tumor, anatomical areas affected, clinical appearance of the metastasis, timing of CM detection with respect to primary tumor diagnosis, presentation of the metastasis, number of skin lesions, therapeutic plan, and existence of metastasis to other regions).

In the statistical analysis, we applied the Kolmogorov-Smirnov test to check the normality of distribution, then used the Student’s t-test (unpaired samples) or Mann-Whitney test (non-normal distribution) to compare the numerical variables between groups; analysis of variance (ANOVA) to compare the numerical variables between more than two groups, with Bonferroni or nonparametric Kruskal-Wallis post-hoc tests; and the chi-square test or Fisher’s exact test for categorical variables. P-values below 0.05 were considered significant. All statistical analyses were performed using Statistical Package for the Social Sciences (SPSS) version 18.0 (SPSS Inc., Chicago, USA).

Our study followed the standards laid down in the Declaration of Helsinki and updated in Edinburgh in 2000; the Council of Europe Convention on Human Rights and Biomedicine; the United Nations Educational, Scientific and Cultural Organization (UNESCO) Universal Declaration on Bioethics and Human Rights; and the requirements of Spanish law concerning biomedical research, personal data protection, and bioethics. Per the exception specified in the circular letter 15/2002 of the Spanish Agency of Medicines and Medical Devices, we considered it unnecessary to obtain informed consent as all personal health data was codified in the database, and all patients, therefore, remained anonymous. The protocol was approved by the ethics committee of Alicante University General Hospital, Alicante, Spain.

## Results

Of the 119 patients with CM who were seen during the study period, we excluded 60 patients with melanoma, 11 with hematological neoplasms, and 11 with incomplete data, leaving a study sample of 37 patients. This sample included 20 men (54%) and 17 women (46%), and the mean age was 62 ± 15 years. The number of clinical images obtained per patient ranged from 1 to 16, with most patients (65%) having fewer than four photos.

The median time elapsed between tumor diagnosis and CM detection was 10 (0-72) months, and the mean follow-up time until death was five (0-204) months. Five patients, in treatment with chemotherapy, were still alive when we conducted our review, with a mean follow-up time of 20 (10-60) months. The primary tumor was known in 35 cases (95%) and unknown in two cases (5%). CM was the first sign of the primary tumor in nine cases (24%).

**Figure 1:**
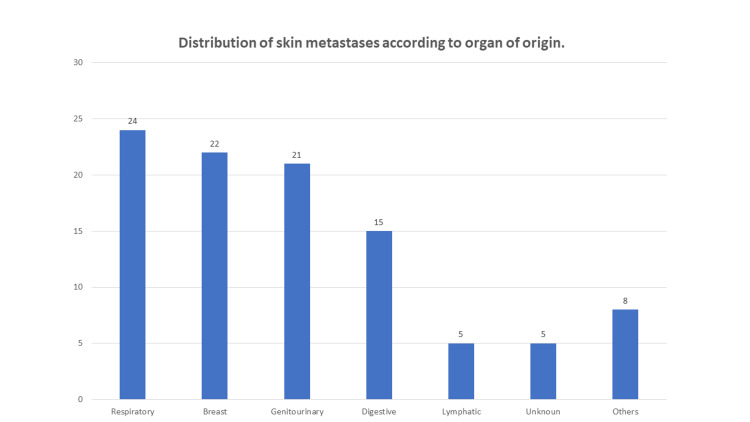
Distribution of cutaneous metastases according to organ system of primary tumor site

Timing of CM detection was classified as early in three patients (8%), synchronous in 12 (32%), and metachronous in 22 (60%). The primary tumor was stage IV in all patients. When we analyzed the source of CM by organ system, we found that most began in the respiratory system (24%), followed by breast (22%) and the genitourinary apparatus (21%) (Figure 1). In terms of specific organs, the largest proportion of CM resulted from lung carcinoma (24%) followed by breast carcinoma (22%) and bladder carcinoma (11%).

**Figure 2:**
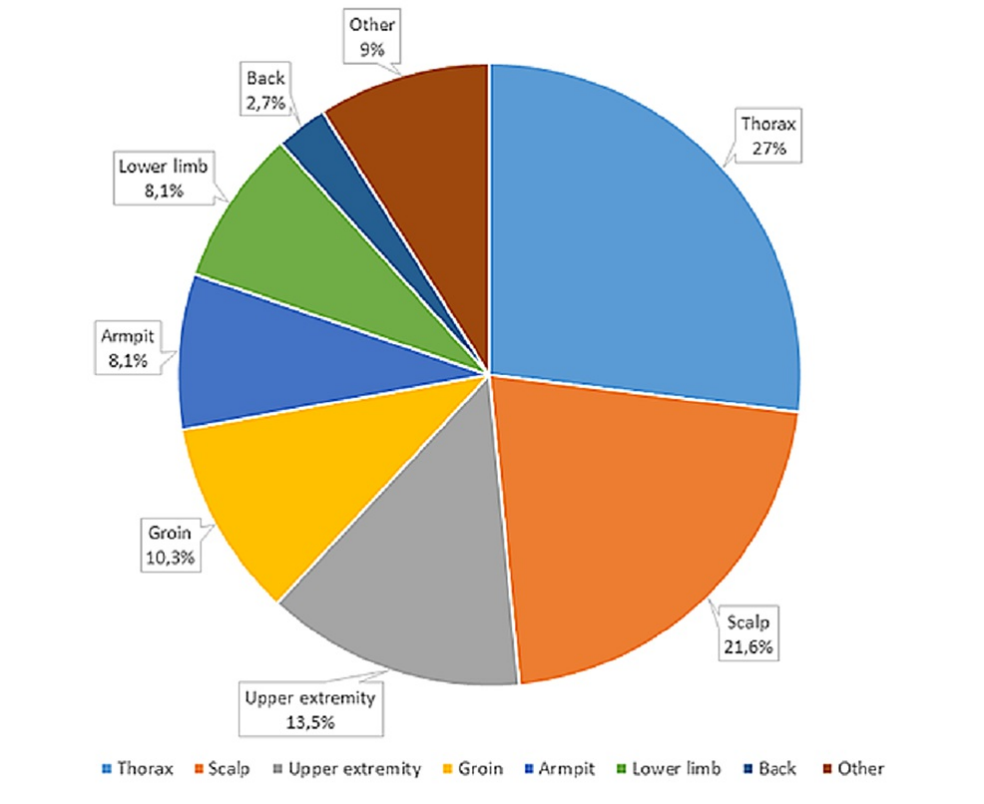
Most frequent anatomical locations of cutaneous metastases

Metastatic lesions were solitary in 57% of cases and multiple in 43%, affecting a single anatomical area in 87% of cases and several areas in 13%. The most frequent anatomical locations were the chest and scalp (Figure 2), though our sample included particular forms such as a “clown nose” secondary to lung cancer and an acral CM secondary to clear cell renal cell carcinoma.

**Figure 3:**
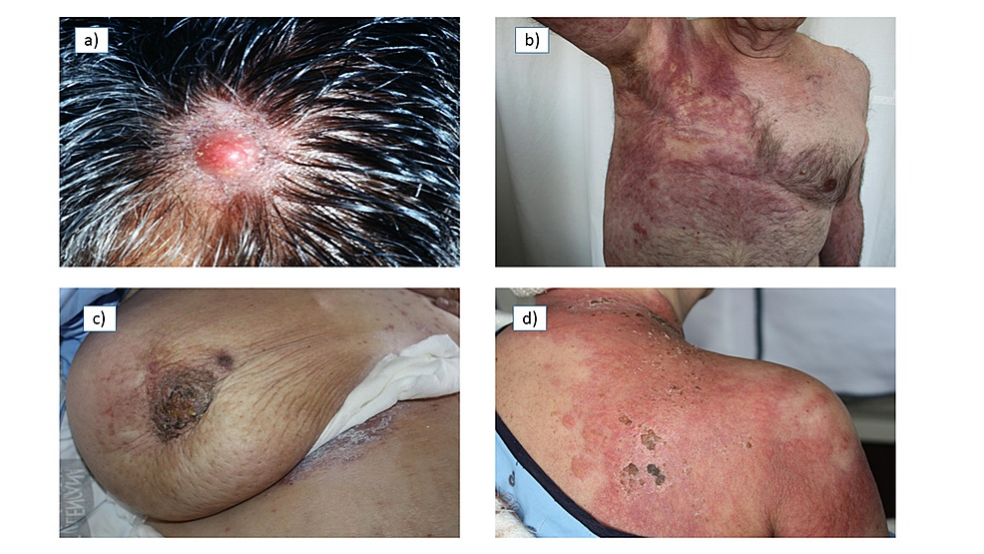
Images of scalp metastasis secondary to squamous cell carcinoma of the lung (a); metastasis in a "shell" pattern secondary to infiltrating ductal carcinoma of the breast in a male (b); metastasis with "orange peel" pattern secondary to infiltrating ductal carcinoma of the breast (c); and metastasis with "erysipeloid" pattern secondary to infiltrating ductal carcinoma of the breast (d)

Figure 3 presents a few examples of CM images.

**Figure 4:**
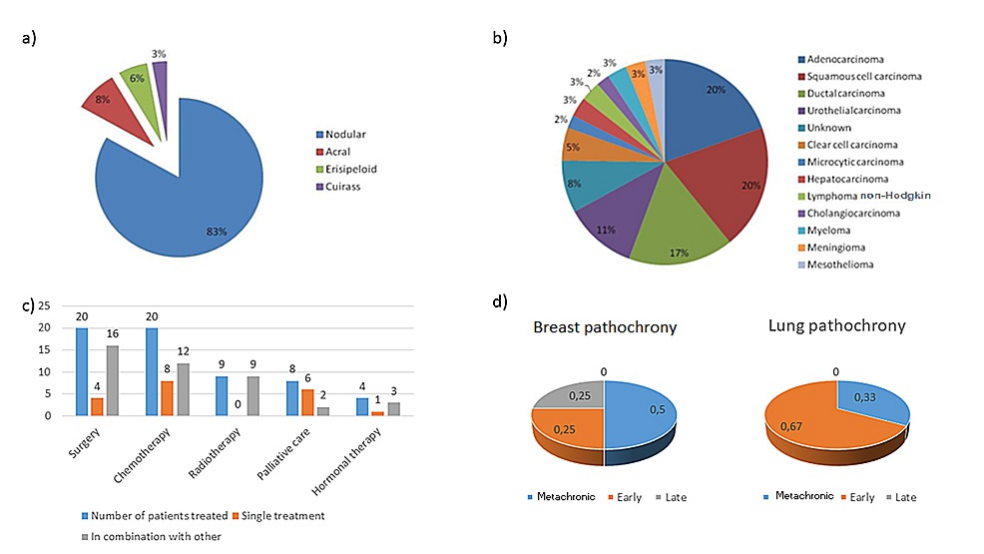
(a) Dermatological patterns of cutaneous metastasis. (b) Anatomopathological diagnosis of skin metastases. (c) Therapeutic management of the cases. (d) Comparison of the pathochrony of the appearance of skin metastases in their most frequent origins

In 100% of cases, dermatological examination results suggested that the lesions constituted CM of a primary tumor, while pathology findings indicated primary tumor origin in 97% of cases. The appearance of the CM was most frequently nodular (Figure 4, Panel a), and in 22% of cases, these nodules were ulcerated.

Figure 4 (Panel b) shows the final pathological diagnosis regarding the source of the CM. Breast carcinoma accounted for 21% of the total, specifically invasive ductal carcinoma in 16%. Lung cancer accounted for 25% of cases, with equal percentages (10.8%) of adenocarcinoma and squamous cell carcinoma.

The main immunohistochemical markers used were one or more of the following: thyroid transcription factor-1 (TTF1), CD45, CD79a, CD3, CD43, CD30, CK7, CK20, CK34, CK19, Ki69, estrogen receptors, progesterone receptors, human epidermal growth factor receptor 2 (HER2), B-cell lymphoma (Bcl), and cadherin, corresponding to the cell line of the primary tumors.

Figure 4 (Panel c) shows the distribution of therapeutic management, which in most cases consisted of surgery combined with chemotherapy and/or radiotherapy, hormone treatment, or palliative care. At the time of diagnosis, CM was not accompanied by other metastases in 27% of cases, whereas 46% of included patients had metastases affecting multiple systems, 5% affecting the bones, 3% affecting the lungs, 3% affecting the retroperitoneum, and 3% affecting the brain.

Given the high frequency of breast and lung carcinoma, we compared some clinical variables between the two tumors, between breast carcinoma and the remaining tumors, and between lung carcinoma and the remaining tumors. The main difference between lung and breast carcinoma was the predominance of one or the other according to patient sex, with lung tumors significantly more common in men (89%) and breast tumors more common in women (88%). We found no differences in other clinical variables or the timing of detection between CM of lung and breast tumors (Figure 4, Panel d).

When we compared CM of breast carcinoma with the remaining tumors, we found significantly more plaque-like lesions and fewer nodular lesions (p < 0.05), and no other significant differences (Table 1).

**Table 1:** Pathochronology, timing, and presentation of cutaneous metastases of breast cancer compared with other tumors

Pathochrony	Breast Median (min-max)	Other Tumors Median (min-max)	p-value
Time from tumor diagnosis to appearance of metastases (months)	7.5 (0-72)	10 (0-48)	p: 0.08
Follow-up time to exitus letalis (months)	36 (6-204)	3 (0-120)	p: 0.012
Time to Appearance	Breast N (%)	Other Tumors N (%)	Chi-Square
Early	2 (25%)	1 (3.4%)	p: 0.08
Synchronic	2 (25%)	10 (34%)	p: 0.07
Metachronic	4 (50%)	29 (62.1%)	p: 0.09
Mode of Presentation	Breast N (%)	Other Tumors N (%)	Chi-Square
Nodule	5 (62.5%)	27 (93.1%)	p: 0.03
Mass	3 (37.5%)	2 (6.9%)	p: 0.02

Compared with the rest of primary tumors, in CM of lung cancer, we found significantly shorter time from tumor diagnosis to detection of metastasis and shorter follow-up time in surviving patients (p < 0.01 in both cases); more synchronous, fewer early and fewer metachronous cases (p < 0.05); and more anatomical areas affected (p < 0.01) (Table 2).

**Table 2:** Pathochronology, timing, location, and presentation of cutaneous metastases of lung cancer compared with other tumors

Pathochrony	Lung (n = 9) (Mean ± SD)	Other (n = 28) Tumors (Mean ± SD)	p-value
Age	62 ± 12.9	63 ± 16.3	p: 0.08
Time from tumor diagnosis to appearance of metastases (months)	3 ± 5	14 ± 17	p: 0.007
Follow-up time to exitus letalis (months)	24 ± 47.2	21 ± 41.5	p: 0.09
Follow-up time in non-deceased patients	12 ± 9.7	102 ± 8.4	p: 0.005
Time to Appearance	Lung N (%)	Other Tumors N (%)	Chi-Square/Fisher‘s Exact
Early	0 (0%)	3 (10.7%)	p: 0.12
Synchronic	6 (66.7%)	6 (21.4%)	p: 0.10
Metachronous	3 (33.3%)	19 (67.9%)	p: 0.07
Areas of Location	Lung N (%)	Other Tumors N (%)	Chi-Square/Fisher‘s Exact
One anatomical area	5 (55.6%)	27 (96.4%)	p: 0.009
Several anatomical areas	4 (44.4%)	1 (3.6%)	p: 0.03
Mode of Presentation	Lung N (%)	Other Tumors N (%)	Chi-Square/Fisher‘s Exact
Nodule	8 (88.9%)	24 (85.7%)	p: 0.07
Mass	1 (11.1%)	4 (14.3%)	p: 0.10

Most cases of CM from the breast and the lung coexisted with metastases affecting other systems. We compared the location of breast and lung cancer metastases (other than CM) with those of other tumors, finding interesting though nonsignificant results: Breast tumors were far less likely to result in multisystemic metastases (in addition to CM) compared with other tumors, while this difference was less clear with lung cancer. Indeed, additional metastases were most frequently multisystemic in cases of lung cancer CM.

## Discussion

The low prevalence of CM explains the apparently small size of our study sample, which was nonetheless representative, thanks to the long study period. The largest series of CM in the literature include no more than 100 to 200 patients [14-16]. Regarding mean age at diagnosis, our findings coincide with those of the most recent series [17-19]. The proportion of men and women in our sample was very similar, with a slightly higher percentage of men unlike in previous studies [17,20].

CM is sometimes the first manifestation of a tumor; this was the case for three patients in our sample (8%). Meanwhile, in a third of cases, the primary neoplasm was diagnosed at the same time as the CM, during the preliminary study of the tumor. CM detection was most frequently metachronous, meaning there was a latency period between primary tumor diagnosis and the occurrence of skin lesions.

One finding indicating poor prognosis was the number of deceased patients: 32 of 37. The mean follow-up time from CM diagnosis to death was 22 months, and several patients died less than five months after diagnosis, reflecting the fact that CM is an indicator of distant spread. Primary tumors in our study most frequently affected the lung (24%) and breast (22%), in concordance with the most recent reviews [17]. In various studies, breast and lung neoplasms were the most frequent types in women and men, respectively; this pattern, no doubt associated with the high prevalence of both cancers, was repeated in our study.

The most common locations of CM in our series were the head and chest (including the armpits). A large proportion of lesions were located in the groin. This distribution correlates with the draining lymph nodes and vascularization of the main primary tumors studied.

In line with other studies [1,8], the most frequent presentation of CM was a solitary nodule, although breast cancer CM was more likely to appear as a plaque, in some cases with characteristic patterns such as carcinoma erysipeloides or “orange peel.” Despite the limited sample size, we found an interesting number of special presentations such as acral lesions secondary to carcinoma of the kidney, cervix, and breast as well as a “clown nose” lesion secondary to lung cancer. In only two cases, the histological pattern of the lesions was nonspecific, corresponding to the neoplasms of unknown origin. Analysis of the remaining cases revealed predominant patterns for CM of breast and lung tumors.

Given the high prevalence of breast and lung CM, we performed exhaustive comparisons between the two and between each of them and the remaining tumors. On comparing the breast with the remaining primary tumor sites, one of the most significant findings was the presentation in plaque form, as described above. In addition, follow-up time until death in breast cancer patients was twice of that observed in the rest of the patients, though this difference was nonsignificant owing to the small sample size. In contrast, follow-up time until death as well as the time elapsed from primary tumor diagnosis until CM detection was significantly shorter in lung cancer patients compared with the remaining patients. Lung cancer CM tended to be diagnosed at the same time as the tumor (on the same day or a few days later), and lesions most often appeared in several anatomical areas, revealing disseminated disease from the beginning. These aspects confirm the highly aggressive nature of lung neoplasms, which progress rapidly to advanced stages and have poor survival outcomes regardless of the therapeutic approach.

A total of 27 patients were diagnosed with metastases affecting other systems (in addition to CM). Most of these cases were multisystemic, affecting more than one organ, although in the breast cancer patients, additional metastases mostly appeared in the lymph nodes only. This is most likely attributable to the advances achieved in the early diagnosis of breast tumors as a result of screening techniques and public awareness of the importance of regular self-examination. Lung cancer, on the other hand, is far more prone to multisystemic metastasis, which again confirms the aggressive nature of this malignancy.

Our study has some potential limitations, not least the relatively low number of CM cases. Nonetheless, our series spanned a long period of time (18 years) and is characteristically similar to the published series with more cases [5,11,14]. Given the low incidence of CM, only a multicenter study could include a large number of patients. One strength of our study is the routine performance of biopsies, which enabled us to analyze lineage wherever CM was examined, as well as the protocolized collection of all clinical variables.

## Conclusions

The results of our study suggest that CM tends to affect patients aged 60 and older and that the most frequent primary tumor site is the lung in men and the breast in women. CM typically occurs on the chest and head and has a nodular appearance, although these factors vary depending on the primary neoplasm. We conclude that CM is a poor prognostic sign associated with poor survival but related to the primary tumor prognosis.
